# The Crashing Obese Patient

**DOI:** 10.5811/westjem.2018.12.41085

**Published:** 2019-02-06

**Authors:** Brian K. Parker, Sara Manning, Michael E. Winters

**Affiliations:** * University of Texas San Antonio, Department of Emergency Medicine, San Antonio, Texas; † University of Maryland School of Medicine, Department of Emergency Medicine, Baltimore, Maryland

## Abstract

Emergency physicians (EP) frequently resuscitate and manage critically ill patients. Resuscitation of the crashing obese patient presents a unique challenge for even the most skilled physician. Changes in anatomy, metabolic demand, cardiopulmonary reserve, ventilation, circulation, and pharmacokinetics require special consideration. This article focuses on critical components in the resuscitation of the crashing obese patient in the emergency department, namely intubation, mechanical ventilation, circulatory resuscitation, and pharmacotherapy. To minimize morbidity and mortality, it is imperative that the EP be familiar with the pearls and pitfalls discussed within this article.

## INTRODUCTION

Obesity has become one of the nation’s leading public health crises.^1^ In fact, more than one-third of the adult population of the United States (U.S.) is now considered obese.^2^ Obesity is typically defined as a body mass index (BMI) greater than 30 kilograms per square meter (kg/m^2^). People with a BMI greater than 40 kg/m^2^ are classified as morbidly obese.^3^ As BMI increases, so does the incidence of significant comorbid conditions such as diabetes, obstructive sleep apnea, hypertension, and dyslipidemia. In addition, obesity induces a number of anatomic and physiologic changes that affect resuscitation and emergency department (ED) management.

The emergency physician (EP) is frequently called upon to resuscitate and manage critically ill patients. The obese patient whose condition is unstable, rapidly changing, and requires emergent resuscitation, the so-called “crashing” obese patient, presents a unique challenge for even the most skilled EP. Changes in anatomy, metabolic demand, cardiopulmonary reserve, ventilation, circulation, and pharmacokinetics require special consideration. This article focuses on critical components in the resuscitation of the crashing obese ED patient, namely rapid sequence intubation, mechanical ventilation, circulatory resuscitation, and pharmacotherapy. To minimize morbidity and mortality, it is imperative that the EP be familiar with the pearls and pitfalls discussed in this article.

## ALTERATIONS IN RESPIRATORY PHYSIOLOGY

The respiratory system of the obese patient undergoes several anatomic and physiologic alterations that affect emergent airway management and initiation of mechanical ventilation. Anatomically, obese patients have an increased neck circumference due to excess cervical adipose tissue. Increased neck circumference is strongly associated with the upper airway collapse observed in obstructive sleep apnea.^4^ Additionally, increased soft tissue deposition in the relatively closed space of the oropharyngeal cavity leads to pharyngeal airway narrowing.^5^ As observed in sleep, loss of neuronal compensation in the setting of sedation with or without paralytic can lead to upper airway collapse. Increased neck circumference as well as dorsocervical fat deposition can limit neck extension. While it remains unclear if obesity is an independent risk factor for a difficult airway, obesity and its associated conditions are considered in several commonly used scoring systems to assess for potentially difficult intubations, including the Wilson scoring system, LEMON (Look-Evaluate-Mallampati-Obstruction-Neck mobility), and the HEAVEN (Hypoxemia, Extremes of size, Anatomic challenges, Vomit/blood/fluid, Exsanguination/anemia, Neck mobility issues) criteria.^6^–^8^ Rapid access to a surgical airway can be limited when landmarks are obscured in a short, obese neck. Some recommend initial sharp dissection followed by palpation within the incision to facilitate landmark identification.^9^ Overall, obesity and its associated anatomic changes should alert the EP to the possibility of a difficult airway and prompt appropriate planning and back-up.

Physiologically, obese patients have markedly decreased lung volumes. In fact, for each unit increase in BMI, functional residual capacity (FRC), expiratory reserve volume, vital capacity, total lung capacity, and residual capacity decrease 0.5% to 5%.^10^ Of these changes in lung volumes, the reduction in FRC is perhaps the most important, as further decreases lead to the closure of small airways and an increase in airway resistance.^1^ Reduction in FRC is an important contributor to the marked limitation in safe apnea time in the obese. Increased airway resistance results in under-ventilated areas of lung, atelectasis, and intrapulmonary shunting.^1^ Decreased lung volumes also reduce lung compliance in the obese patient. In addition to decreased lung volumes, decreased lung compliance, increased airway resistance, and intrapulmonary shunting, obese patients also develop ventilation-perfusion (V/Q) mismatch due to the fact that their upper lung zones are aerated preferentially, whereas lower lung zones are perfused preferentially.^1^ Finally, chest wall compliance is reduced due to the increase in adipose tissue in the thoracic cage. All of these alterations in respiratory physiology can be worsened when the obese patient is placed in the supine position.

As a result of these physiologic changes, it is not surprising that oxygen consumption and the work of breathing (WOB) are significantly increased in the obese patient.^11^ Oxygen consumption is approximately 1.5 times higher in the obese patient than in the non-obese patient.^11^ Due to the increase in oxygen consumption and WOB, obese patients produce more carbon dioxide than non-obese patients.^12^ To compensate, the obese patient adopts a rapid, shallow breathing pattern. In fact, normal spontaneous respiratory rates in the morbidly obese patient range from 15–21 breaths per minute compared with 10–12 breaths per minute in non-obese patients.^13^,^14^

Overall, these anatomic and physiologic alterations in respiratory physiology lead to a marked decrease in pulmonary reserve.^1^ Decreased pulmonary reserve predisposes the patient to the rapid onset of hypoxemia during rapid sequence intubation (RSI), which can result in peri-intubation cardiac arrest.

## INTUBATION

In critically ill obese patients, intubation is a high-risk procedure that can be fraught with peril. As discussed in the preceding section, obese patients have very little cardiopulmonary reserve and can desaturate rapidly to critical oxygen levels during intubation. Numerous studies have highlighted obesity as a risk factor for difficult intubation.^15^–^18^ De Jong and colleagues reported an increased incidence of difficult intubation in obese patients.^19^ They found that an elevated Mallampati score, limited mouth opening, reduced cervical mobility, the presence of obstructive sleep apnea, and severe hypoxemia were associated with difficult intubation.^13^,^19^ Additional factors that have been shown to predict difficult intubation in obese patients include a short neck, a thick neck, diabetes mellitus, and abnormal upper teeth.^1^,^20^,^21^ Given the challenges of airway management in the obese patient, it is crucial for the EP to optimize intubation conditions to reduce the risk of poor outcome.

### Preoxygenation

Critically ill patients undergoing RSI should be preoxygenated adequately prior to intubation in order to prolong the time to reach critical oxygen saturation thresholds during apnea. The primary goal of preoxygenation is to create an oxygen reservoir by replacing nitrogen within the FRC with oxygen.^1^ Common methods of preoxygenation include the use of a face-mask (FM) with 100% fractional inspired oxygen concentration (FiO_2_), bag-mask ventilation (BMV), noninvasive positive pressure ventilation (NIV), and high-flow nasal cannula (HFNC) devices. Often, the traditional methods of preoxygenation using a FM or BMV are insufficient in the critically ill obese patient.^13^ But NIV can be beneficial and is the preoxygenation method preferred by many.^13^ The application of continuous positive airway pressure (CPAP) at 10 centimeter (cm) H_2_O has been shown to reduce atelectasis, improve oxygenation, and increase apnea time without hypoxemia in the obese patient undergoing surgery.^13^,^22^,^23^ Bilevel positive airway pressure (BPAP) can also be used to preoxygenate obese patients, although it is less well studied than CPAP.^1^ Compared with the use of a FM with 100% FiO_2_, BPAP improves oxygen saturation readings prior to intubation.^1^,^24^ When clinically feasible, CPAP or BPAP should be maintained for at least five minutes during the preoxygenation period.^25^ HFNC devices can be considered for the obese patient; however, the minimal positive pressure delivered by HFNC devices can be expected to have little impact on FRC, and evidence supporting their benefit in preoxygenation prior to RSI is limited.^13^

### Patient Positioning

Proper positioning is critical for success in both preoxygenation and intubation of the obese patient. Given the alterations in respiratory physiology, obese patients should be placed in either a semirecumbent (head of the bed elevated to 25 degrees) or a sitting position during preoxygenation.^1^,^13^ The upright or semirecumbent position may decrease air trapping, decrease atelectasis, and improve oxygen saturation prior to intubation.^13^,^26^ Similar to the optimal position for preoxygenation, obese patients should be placed in a head up or ramped position to optimize the laryngoscopic view for intubation (Figure).^1^,^27^–^29^ To ensure proper position, the EP should align the patient’s sternal notch with his or her external auditory meatus.^1^,^28^,^29^

### Medication Dosing

Improper dosing of RSI medications can cause significant patient discomfort and may increase the incidence of complications during intubation. Several recent studies demonstrated that obese patients often receive inappropriate doses of sedative and paralytic medications during RSI.^30^–^32^ Bhat and colleagues demonstrated that obese patients were more likely to be underdosed with both etomidate and succinylcholine during RSI.^32^ It is therefore important for the EP to be knowledgeable about the proper dosing of medications commonly used during RSI (Table). Medications dosed on* total* body weight include etomidate and succinylcholine, whereas propofol and the nondepolarizing neuromuscular blocking medications (e.g., rocuronium) are dosed on *ideal* body weight.^1^,^21^ Ketamine is dosed on lean body mass.^1^,^21^

### Laryngoscopy

It is wise for the EP to consider each intubation of an obese patient as a difficult intubation. As such, adequate preparation is of paramount importance. In addition to the equipment needed for direct laryngoscopy, advanced airway equipment (e.g., supraglottic airway, video laryngoscope, gum elastic bougie, surgical airway equipment) should be placed at the bedside. Video laryngoscopy may be preferred over direct laryngoscopy in the obese patient.^1^,^33^,^34^ For patients who require BMV during intubation attempts, recall that obesity is a risk factor for difficult BMV.^13^,^35^ The use of an oral or nasal airway, a two-handed jaw thrust, or a two-person technique can improve the efficacy of BMV.^1^,^21^

## MECHANICAL VENTILATION

Initiation of mechanical ventilation in the intubated obese ED patient can be challenging. Improper ventilator settings can lead rapidly to respiratory or hemodynamic deterioration and increased morbidity and mortality. Similar to the mechanical ventilation of non-obese patients, important ventilator settings for the obese patient include ventilator mode, respiratory rate, positive end-expiratory pressure (PEEP), and, in volume-controlled modes, tidal volume.

The two most common modes of mechanical ventilation used in the obese patient are volume-controlled ventilation (VCV) and pressure-controlled ventilation (PCV).^36^ To date, the superiority of one mode over the other has not been demonstrated in the literature.^13^ Notwithstanding, some clinicians prefer PCV, as the decelerating waveform may improve distribution of airflow to the alveoli.^13^

The benefits of a low-tidal-volume (6–8 milliliters per kilogram [ml/kg]) ventilation strategy in patients with acute respiratory distress syndrome (ARDS) have been well established.^37^,^38^ In recent years, the use of low-tidal-volume ventilation has also been recommended for patients without ARDS.^39^–^41^ Importantly, the tidal volume must be calculated using *ideal* body weight rather than total body weight. This is especially important for the intubated obese patient, for whom the use of total body weight to determine the tidal volume can lead to injurious lung volumes, barotrauma, and ventilator-induced lung injury.

As previously discussed, obese patients produce excessive amounts of carbon dioxide due to increased metabolic demand, increased oxygen consumption, and increased WOB.^11^–^14^ As a result, they adopt a rapid, shallow breathing pattern and have a normal respiratory rate that ranges from 15–21 breaths per minute.^13^,^14^ When setting the ventilator, it is important to account for this altered physiology and initially set a higher respiratory rate than for the non-obese patient.^13^

Obese patients demonstrate improved respiratory mechanics and alveolar recruitment when provided with PEEP.^1^,^13^,^42^ PEEP reverses airflow limitations and helps to prevent alveolar derecruitment caused by the decrease in FRC.^1^,^13^ Importantly, the optimal level of PEEP in ventilated obese patients remains uncertain.^13^ They might benefit from a higher initial PEEP setting (i.e., 10 cm H_2_O) in contrast to non-obese patients, who are commonly started on lower levels of PEEP (i.e., 5 cm H_2_O).^13^,^43^,^44^ The initial PEEP setting in the individual obese patient should also take into account the anticipated hemodynamic effects when PEEP exceeds extant intrathoracic pressure, including decreases in venous return, right ventricular output, and pulmonary perfusion. Expiratory flow limitation observed in the obese can result in an auto-PEEP phenomenon. In that event, extrinsic PEEP should be set at two-thirds intrinsic PEEP.^13^

Finally, the ventilated obese patient should be placed in a reverse Trendelenburg or sitting position.^1^ Similar to optimal patient positioning for preoxygenation and RSI, the reverse Trendelenburg or sitting position reduces intrathoracic pressure, reduces atelectasis, improves V/Q mismatch, decreases the incidence of hypoxemia, and may improve the laryngoscopic view.^27^,^28^

## ALTERATIONS IN CIRCULATORY PHYSIOLOGY

Similar to the respiratory system, the physiology of the circulatory system is altered in obese people. Alterations in circulatory physiology were first reported in 1964 by Alexander, who described the linear relationship between a patient’s weight and total blood volume.^45^ Greater total blood volume increases stroke volume, which ultimately leads to an increase in preload and myocardial wall tension. Ferraro and colleagues demonstrated that these physiologic changes cause an eccentric left ventricular hypertrophy, impaired ventricular relaxation, and diastolic dysfunction.^46^ Diastolic dysfunction eventually leads to systolic dysfunction, pulmonary hypertension, and right ventricular hypertrophy. Together, these physiologic changes have been termed the “obesity cardiomyopathy syndrome.”^47^

For the majority of ED patients, initial assessment of the circulatory system begins with a noninvasive blood pressure measurement. Importantly, standard blood pressure cuffs are often too narrow and too short for the obese patient. In many cases, this leads to an overestimation of blood pressure.^48^,^49^ If the obese patient appears moribund, demonstrates signs of poor perfusion (i.e., cool and mottled skin), or is critically injured, the EP should consider early placement of an invasive arterial line to accurately determine the mean arterial blood pressure.^50^

Intravenous (IV) fluid administration is one of the most common interventions in critically ill ED patients. Unfortunately, obesity has been shown to be an independent risk factor for difficult IV access.^51^–^53^ The causes of difficult IV access in the obese patient are likely multifactorial and include increased adipose tissue, increased tissue edema, and smaller vein caliber. Alternatives to peripheral IV access include the intraosseous route and central venous access. Importantly, these alternatives also have limitations and complications in the obese patient. Kehrl and colleagues demonstrated that a standard 25 millimeter (mm)- intraosseous needle might not be long enough for patients with a BMI greater than 43 kg/m^2^.^54^ In these severely obese patients, the EP should consider using an extended 45-mm intraosseous needle.^54^ However, in a study of out-of-hospital cardiac arrest, Kawano and colleagues demonstrated worse patient outcomes in those who had intraosseous vascular access compared with those who had IV access.^55^

Ultrasound guidance is rapidly becoming an essential tool in scenarios involving difficult IV access.^56^ The use of longer catheters reduces the rate of IV dislodgement,^56^ and ultrasound guidance during cannulation has a higher successful rate than the standard blind approach.^57^ Additionally, Au et al. demonstrated that the use of ultrasound guidance for IV access reduced the need for central venous access.^58^

Placement of a central venous line in an obese patient can be problematic. Risks associated with central venous access in the obese patient include inability to place the line, the need to use extended-length needles to assist with cannulation, and higher infection rates.^59^–^61^ Although many physicians use a landmark approach to central venous access, this method is not reliable in obese patients.^62^,^63^ Furthermore, traditional depths of insertion are often too shallow in the obese patient.^61^,^64^ Although EPs are trained in ultrasound-guided cannulation of the central veins, up to 40% of EDs in the U.S. do not have bedside ultrasound available.^65^ Whenever possible, the EP should use ultrasound to guide central venous cannulation of the femoral, internal jugular, and subclavian veins.^65^

For patients in cardiac arrest, the delivery of high-quality cardiopulmonary resuscitation (CPR) is crucial to achieve return of spontaneous circulation. Critical components of high-quality CPR are compressions delivered at the proper rate and depth and allowing full recoil of the chest between compressions.^66^ Current international guidelines for the resuscitation of adult patients in cardiac arrest recommend placement of the hands on the lower half of the sternum during CPR.^66^–^68^ Given the increased adipose tissue of the chest wall and a more cephalad displacement of the diaphragm in obese patients, this hand location might not be the optimal position for CPR in obesity. Lee and colleagues found that the optimal hand position for CPR in obese patients might be slightly more cephalad than in non-obese patients.^67^

## PHARMACOTHERAPY

As highlighted in the preceding intubation section, medication dosing in the obese patient is challenging. Importantly, almost all dosing recommendations have been developed for non-obese patients and are then extrapolated to the obese population. This extrapolation can lead to dosing errors and result in medication toxicity or treatment failure. Proper medication dosing is determined by many factors. Perhaps the most important one is the lipophilicity of the medication. In general, when a medication is highly lipophilic, it rapidly distributes to the peripheral tissues and should be dosed based on total body weight. In contrast, when a medication is hydrophilic, the volume of distribution is lower, so the dose should be based on ideal or adjusted body weight. An additional factor that affects medication dosing is renal function. If a medication is cleared by the kidney, it should be dosed on actual creatinine clearance rather than calculated creatinine clearance.^69^ In the obese patient, the EP should pay special attention to cardiovascular, sedative, antimicrobial, and anticoagulant medications.

### Cardiovascular Medications

Beta (β)-adrenergic receptor blockers, calcium channel blockers, digoxin, lidocaine, and procainamide are commonly administered cardiovascular medications in the ED. β-Adrenergic receptor blockers, digoxin, and procainamide are relatively hydrophilic medications and should be dosed on ideal body weight, whereas calcium channel blockers are more lipophilic and should be dosed based on total body weight. Vasoactive medications (e.g., norepinephrine, epinephrine, dobutamine) do not require dosing adjustments in the obese patient.

### Sedative Medications

Sedative medications are used frequently in the ED for post-intubation sedation, procedural sedation, severe agitation, and induction for intubation. Sedatives are generally highly lipophilic medications that can have prolonged half-lives in the obese patient. To prevent accidental oversedation, the initial dose of a sedative should be based on ideal body weight, with subsequent doses based on the patient’s response and anticipated duration of treatment. The EP should use extra caution with benzodiazepines in the obese patient. When given via continuous infusion, benzodiazepines (along with the analgesic fentanyl) can have an extremely long duration of action.^70^

### Antimicrobial Medications

Given the emphasis on early recognition of sepsis and early administration of broad-spectrum antimicrobial agents, it is imperative to dose them correctly in the obese patient. Fuller and colleagues demonstrated an eightfold increase in the likelihood of underdosing of vancomycin for every 10-kg increase in body weight.^71^ For vancomycin, total body weight should be used to determine the proper initial loading dose.^72^ For penicillins, cephalosporins, and carbepenems, the EP should use the higher end of dosing recommendations. In contrast to these agents, the dose of aminoglycosides should be calculated based on ideal body weight. If the patient has a total body weight that is more than 130% of his or her ideal body weight, then adjusted body weight should be used to calculate the aminoglycoside dose.^73^

### Anticoagulant Medications

Obese patients and those with metabolic syndrome are at increased risk for venous thromboembolic events (VTE).^74^–^77^ Anticoagulant medications used to treat thromboembolism are considered high-risk medications; therefore, proper dosing, especially in the obese patient, is imperative.^78^ Low-molecular-weight heparin (LMWH) is commonly used to treat VTE and is dosed at 1 mg/kg/day. Some LMWH formulations have maximum dosing recommendations, which may lead to subtherapeutic levels in the obese patient.^79^ If the obese patient’s total body weight exceeds 190 kg, anti-Xa levels should be monitored to ensure appropriate levels of anticoagulation.^80^ Unfractionated heparin could be used if LMWH is not available, but LMWH has been shown to be at least equivalent in head-to-head comparisons, with less frequent dosing and less total volume infused.^81^,^82^^X^ To date, no large, randomized controlled trials have evaluated the use of newer, direct oral anticoagulants in the obese patient. If these medications are being considered for treatment of VTE in an obese ED patient, the EP should consider consulting with a pharmacist for dosing recommendations.

## CONCLUSION

Resuscitation of the crashing obese ED patient presents numerous challenges for the EP. Even prior to the development of critical illness, obese patients have alterations in respiratory physiology, circulatory physiology, and pharmacokinetics that significantly affect their ED evaluation and resuscitation. These alterations greatly affect the EP’s approach to rapid sequence intubation; initiation and management of mechanical ventilation; circulatory assessment; vascular access; CPR; and the dosing of critical, high-risk medications. It is our hope that, through the application of the pearls and pitfalls discussed in this article, the EP can minimize morbidity and mortality in this very sick patient population.

## Figures and Tables

**Figure:**
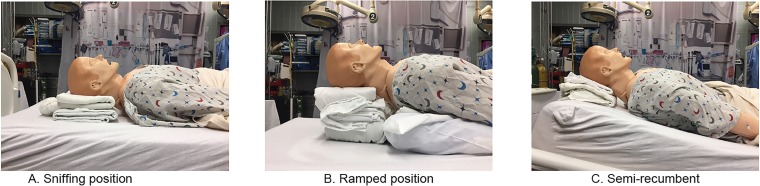
Patient positioning for intubation

**Table:** Weight-based medication dosing.^1^,^16^

Total body weight	Ideal body weight	Lean body mass
Etomidate	Propofol	Ketamine
Succinylcholine	Rocuronium	
Fentanyl	Vecuronium	
Midazolam		
